# How Young Mothers Rely on Kin Networks and Formal Childcare to Avoid Becoming NEET in the Netherlands

**DOI:** 10.3389/fsoc.2021.787532

**Published:** 2022-01-27

**Authors:** Alexander Dicks, Mark Levels, Rolf van der Velden, Melinda C. Mills

**Affiliations:** ^1^ WZB - Berlin Social Science Center, Berlin, Germany; ^2^ Research Centre for Education and the Labor Market, Maastricht University, Maastricht, Netherlands; ^3^ Leverhulme Centre for Demographic Science and Nuffield College, Oxford University, Oxford, United Kingdom

**Keywords:** NEET, kin support, childcare, young mothers, Netherlands

## Abstract

Motherhood is often cited as one of the main reasons for young women to become NEET (not in employment, education, or training). Given the potential long-term negative implications of NEET status, it is important to understand which types of resources can help young mothers to avoid becoming NEET around childbirth. In this paper we investigate how the chances of young mothers to become and stay NEET around the time of first birth are related to the availability and characteristics of members of their social support network, especially partners and grandparents, to assist in childcare. In addition, we consider the local availability of formal childcare. We use population-wide register data from the Netherlands and estimate discrete-time eventhistory models. Our results show that young mothers who are cohabitating or married are less likely to become NEETs than single mothers. We also show that economic activity and relative wage of both young mothers and their partners decreases the likelihood to become NEET and to exit NEET. With respect to the grandparents, we find that having more grandparents live in the immediate vicinity is associated with a lower likelihood to become NEET and a higher likelihood to exit NEET. Furthermore, we find that young mothers with economically inactive parents are more likely to become and less likely to exit NEET. Lastly, we find evidence for crowding-out of informal and formal childcare. Formal and informal childcare sources interact in such a way that the role of either becomes less important as more of the other is available.

## Introduction

In recent decades, women’s labor market participation in Western European countries has dramatically increased. Still, many women leave the labor force after having a child (Joshi et al., 1996; Aisenbrey et al., 2010). Even a temporary retreat from work or education may negatively affect the acquisition of human capital and consequently of occupational status and earnings. This can lead to substantial gender wage gaps that emerge around childbirth^1^ (e.g. Kleven et al., 2019). One group that is not often studied in this context are young mothers and their school-to-work transition. As they most likely have not yet established a stable career or are still in education, prolonged economic inactivity and educational drop-out could mean a significant source of negative late life outcomes for themselves and potentially their children. Young mothers face the additional structural conflict of interest between motherhood and education (Sniekers & van den Brink, 2019). They are more likely ‘not in education, employment, or training’ (NEET) (Statistics Netherlands, 2018; Klug et al., 2019), have achieved less education, less income, and lower employment probability at a later age (Johansen et al., 2020) and a higher welfare dependence at a later age (Gibb et al., 2015). Being NEET is correlate to a myriad of negative outcomes, such as negative feelings, lower life income, and a higher risk of social exclusion and disengagement (e.g. Bynner & Parsons, 2002; Eurofound, 2012; Maguire, 2018; Levels et al., 2022). Therefore, it is important to understand which resources can enable young mothers to complete their education and to (re-)enter the labor force. One such resource are social support and kin networks, and especially grandparental childcare, which are of great importance to mothers in general (e.g., Hank & Buber, 2009; Arpino et al., 2014) and to young mothers in particular (Maguire, 2018; Sniekers & van den Brink, 2019; Ypeij, 2009). Hence, in this study, we will study the relationship between the availability of social support networks and childcare for young mothers’ school-to-work transitions.

The Netherlands provides an interesting case to study the role of kin networks and institutions in childcare. In the past, the male breadwinner model was the most dominant gender role pattern (Clerkx & van IJzendoorn, 1992). This has only partly changed, and childcare is still considered women’s responsibility (Mills et al., 2014; Sniekers & van den Brink, 2019). This is also reflected in Dutch policies and institutions. Rather than providing extensive leave schemes like other European countries, childcare policies in the Netherlands are reflective of the one-and-a-half-earner model with strong employment protection for part-time workers (Lewis et al., 2008). The one-and-a-half earner model is also reflected in formal childcare arrangements. Dutch parents are reluctant to put their children in full-time formal childcare (Portegijs et al., 2006). In addition to concerns about lacking availability, especially in rural areas (Noailly & Visser, 2009), this reluctance might be explained by long held beliefs about its poor quality care (Leitner, 2003). The cost of childcare is another factor. Generally, the cheaper it is, the higher is the labor force participation of women (Connelly, 1992; Zamarro, 2020). Although economic theory assumes that different sources of childcare are perfectly substitutable, this might not be the case if there are concerns about the quality and availability of public childcare provisions (van Ham & Büchel, 2004; Portegijs et al., 2006; Arpino et al., 2014). And although substantial subsidies are available, childcare in the Netherlands is not subsidized if either parent is not working and not in education (and thus NEET). Young mothers are especially affected by this and often struggle to combine motherhood and education or employment (Sniekers & van den Brink, 2019). There is also a considerable social class gap in childcare use. Among poor households, childcare use is less than half than that of rich households (Mills et al., 2014). The combination of short parental leave and low public support for mothers with children under the age of three leaves the Netherlands with one of the widest coverage gaps of early childcare in Europe, which to close needs family support (Saraceno & Keck, 2010; Bordone et al., 2017).

Based on this, one might ask how young mothers can overcome this lack of institutional support (Uunk et al., 2005). In the Dutch policy regime of “familialism by default” (Saraceno & Keck, 2010), especially from maternal grandparents will provide childcare. Indeed, the use of informal care in the Netherlands is very high (Mills et al., 2014) and about half of Dutch parents report having received childcare support from the grandparents (Knijn & Liefbroer, 2006). However, for young mothers whose own parents might not yet be in retirement themselves, access to grandparental childcare might be restricted and depend on other factors and strategies as well. For instance, grandparents and young mothers might coordinate their work schedules to fit their needs (Sniekers & van den Brink, 2019). The relationship of childcare by grandparents and other family members and the labor market activity of mothers has been studied in various ways. Most studies find positive associations between grandparental childcare and labor market participation. But depending on the data and methods used, results may vary. We discuss the results, merits, and downsides of previous studies after the introduction. Supplementary Appendix A1 gives an overview of these studies.

We merge the literature on mother’s labor force participation with the literature on NEET, where we are among the first to use longitudinal data to study NEET (cf. Contini et al., 2019). We aim to understand how the availability of kin support networks and formal childcare contribute to the decision to (a) withdraw from the labor force and education and (b) to reintegrate into the labor force or education. Thereby, we aim to answer the following research questions: To what extent can the characteristics of young women, their parents, partners, and the institutional context explain why some young mothers a) become NEET or b) exit NEET status?

To answer these questions, we analyze population-wide longitudinal register data from the Dutch Social Statistical Database (Bakker et al., 2014). These register data are prospective, so that we can observe the whole school-to-work transition and economic activities of all persons involved and without recall bias. Because the data are collected by institutions and not reported by individuals, there are also no issues with reporting bias or panel attrition. Second, the data enable us to observe the whole population of the Netherlands. Since young mothers are a small group, having population wide data is important. Studies based on most survey data cannot specifically focus on young mothers and often exclude them or other small groups because of sample size issues. In addition to our cohort of interest, we can link their data to data of their extended family and their partners. Most importantly, we can supplement this with detailed geographical data that many studies do not have access to (Compton, 2015). We use discrete-time event history analysis to test the hypotheses derived in the theory section.

### Literature Review

Using a sample of immigrants in France, Dimova and Wolff (2008) showed that spatial distance to grandparents reduced the provision of grandparental childcare and that the provision of grandparental childcare is positively related to the mother’s labor market participation. In a later study, Dimova and Wolff (2011) used the data of ten European countries from SHARE and found strong positive relationships of grandparental care and mother’s labor force participation and involvement. They also consider monetary transfer as a possible mechanism, for which they find no evidence. However, by pooling the data of different countries, the interpretation of the average effect might be unclear and hide between-country differences (Aassve et al., 2012). Also, because SHARE relies on the grandmothers as informants, it lacks some information of the mothers, including their income, and information on daughters-in-law. Using the same data and using retirement eligibility as an instrumental variable, Zamarro (2020) found a significant relationship only in the Netherlands and Greece but not in the other countries included in the data. An instrumental variable approach can be useful to approach a causal interpretation. It works, by estimating the effect from an exogenous variable (here retirement eligibility) on the outcome via the variable of interest (childcare by grandparents) while assuming that the only way the exogenous variable changes the outcome is via the variable of interest. That also implies, that the instrument restricts the sample to grandmothers of working age. Naturally, this approach hinges on having exogenous variation in the first place. Aparicio Fenoll (2020) used shifts in the legal retirement age to instrument for retirement status of grandmothers. The author confirmed the positive relationship between grandmothers’ increased availability due to retirement with the labor force participation of mothers, but only in countries with low family benefits (which according to the author includes the Netherlands). Using the GGS, Aassve et al. (2012) found a positive relationship between childcare by grandmothers and mothers’ labor force participation in Bulgaria, France, Germany, and Hungary. However, after including two instrumental variables, the grandmother being alive and number of siblings, the effect became insignificant in Georgia, Russia, and the Netherlands. While grandparental help is explicitly measured in the Generations and Gender Survey (GGS) data, the study is cross-sectional in design and the authors exclude single mothers, mothers with children under the age of one as well as women who were not survey respondents themselves. Using a family fixed-effects model on longitudinal survey data from the US, Posadas and Vidal-Fernandez (2013) found that grandparental childcare increases the labor force participation of mothers, although not after using the grandmothers death as an instrument. For Italy, Arpino et al. (2014) found that having grandparents care for the children increases the mother’s labor force participation. However, they excluded single mothers because of low case numbers in their survey data. The authors used a similar instrumental variable as Aassve et al. (2012), however extended it to all grandparents being alive, including the parents of the partner. Del Boca (2002) used “having at least one grandparent alive” as a proxy variable for the availability of grandparental childcare and found that this positively correlates with mother’s labor force participation. Similarly, Bratti et al. (2018) found that mothers with children in Italy are more likely to work if their own mother is eligible for retirement. Due to the design of the survey, they had to restrict the sample to cohabitating couples. For Canada, Compton (2015) found that married women with children are more likely to work if their own mother—but not their mother-in-law—lives in close proximity. For single mothers, she did not find an effect on the probability to work but only on working hours. For the US, Compton and Pollak (2014) found that living close to their own mother, and in this case also to their mother-in-law, increases the probability of mothers of young children to work. Also for the US, Krolikowski et al. (2020) showed that after losing a job, young people living in the same neighborhood as their parents benefit from stronger earnings recovery and that this is mainly driven by childcare. In addition to these mostly positive effects, there might also be downsides to relying on grandparents for childcare. Using the German Socio-Economic Panel, García-Morán and Kuehn (2017) found that living closer to their parents and parents-in-law can increase the likelihood to work for mothers between 25 and 50, but that this comes at the cost of lower wages and longer commutes. However, in the data used, it was not possible to distinguish grandparental childcare from childcare by other relatives. Lastly, using data on working women from the European Social Survey, Abendroth et al. (2012) found no significant correlation between the availability of care support from outside the household and mother’s working hours.

All in all, the literature agrees on the positive relationship of grandparental care and labor force participation of mothers. However, there are some limitations with these earlier studies. Few of the listed studies have longitudinal data, hence it is not possible to study exits or entries into the labor force (or education). Another limitation with the used survey data is that the information is gathered from one actor only. Hence, they often lack some key variables for the other actors involved, such as income and economic activity. Few of the existing studies also consider the various forms (formal, informal) of child-care provision at the same time (Blau & Currie, 2006). Yet this is necessary because the availability of formal childcare may substitute or crowd-out informal childcare (Havnes & Mogstad, 2011; Arpino et al., 2014; Bordone et al., 2017). The studies that do consider formal and informal childcare at the same time, mostly rely on between country variation, thereby masking within-country differences of availability of formal childcare.

Another limitation of previous studies is that they mainly explain income and labor force participation but not participation in education. This is understandable, as they mostly did not focus on *young* women. For young people, however, traditional labor market indicators are of limited relevance (Eurofound, 2012). To better capture vulnerable young people on the labor market, the term NEET was coined as a policy definition in the UK in the 1990s (Furlong, 2006). The term is not without criticism and has been criticized to hide within-group differences between those who are ‘merely’ unemployed and those who become inactive long-term (Bynner & Parsons, 2002; Furlong, 2006; Yates & Payne, 2006; Maguire, 2015). The NEET label has also been questioned to be applicable to young mothers, as their decision to become NEET might be voluntary (Tamesberger & Bacher, 2014). However, evidence from qualitative studies showed that many young mothers would like to participate in the labor market or in education–if they could (Maguire, 2018; Sniekers & van den Brink, 2019; Ypeij, 2009).

## Theory

To understand the effect of childbirth on the labor market participation of women, we start from the framework of work-family fit (Voydanoff, 2005). In this framework, women’s decisions regarding the labor market are made by considering the perceived fit of demands and resources in different domains of life. On the one hand, these are demands from work, like working hours and overtime, job demands, and insecurity. These must be balanced with family demands, for example caring for young children. Resources from the family, such as employment and income of the partner and kin support with childcare, can then be used to increase the fit of both domains. We will now lay out the different mechanism for both the partner and the kin support. We follow Begall & Grunow (2015) and also consider the institutional environment in this framework. We extend on Begall & Grunow (2015) by also considering kin support.

### Partners

Partners in the household are the most likely candidates for helping with child rearing. Partners in the nuclear family divide time and effort involved with care work between the two of them. The more a young mother can rely on her partner to help with care work, the less likely it is that she must reduce time on the labour market or in education, and the less likely it is that she becomes NEET. For single mothers, the work-family fit perspective would predict that they cannot rely on the resources of a partner and hence would be less able to achieve a good work-family fit and hence more likely to have to reduce time on the labour market. From this we deduce:

Hypothesis H1: Young mothers who are cohabitating or married are less likely to become NEET and more likely to exit NEET.

If there is a partner present, different perspectives lead to the same expectation regarding the employment and income of the partner. The work-family fit perspective holds that “family-based family support occurs when one spouse serves as the major family provider so that the other spouse may limit work participation to engage more extensively in family activities” (Voydanoff, 2005, p. 9). Family economics and bargaining theories predict that specializations and bargaining power between partners in the household determine the division of unpaid care work and paid work in the household (Becker, 1981; Lundberg & Pollak, 1996; Bittman et al., 2003). This would dictate that the higher the partner’s income from labor is relative to the young mother’s, the higher are the household’s opportunity costs for the partner to not work and the costlier it is for the household to have the partner give up labor earning for child-rearing. This goes vice-versa for the young mother’s earnings compared to the partner’s earnings. Hence, we expect:

Hypothesis H2: The higher the young mother’s income relative to the partner’s, the less likely it is that she becomes NEET and the more likely she is to exit NEET.

### Grandparents

Especially for young mothers in the Netherlands, support from the family is very important (Sniekers & van den Brink, 2019; Ypeij, 2009). Kin support can be understood as the access to resources through the ties of a social network, or social capital (Coleman, 1988; Boisjoly et al., 1995; Portes, 1998). Hence, grandparents who can help raising their grandchild would increase work-family fit. Reduced travel times, lower numbers of adult children, and the fact that grandparents now live longer lives than before has increased the supply of care (Geurts, Van Tilburg, Poortman, and Dykstra, 2014). Especially young mothers often rely on their own parents for childcare (Vandell et al., 2003). Grandparental care has at least three advantages (Portegijs et al., 2006): First, it is often free. Second, because grandparents are often retired, they are very flexible in terms of their time. Fitting the needs of the Dutch part-time culture, grandparental care is also often part-time (Bordone et al., 2017). Third, grandparents are seen as preferred care givers and perceived as more trustworthy and even more qualified than formal care providers. The security of having a trustworthy, familial source of flexible and free childcare if a child falls ill or a parent has to go on a business trip, work overtime, study, or apply for jobs might enhance young mother’s chances of returning to employment or education after having a child even if the available childcare is not used (Compton & Pollak, 2014; Compton, 2015). Yet, the extent to which grandparents can be used as a source for childcare is not the same for everyone. The amount of support they can provide also depends on their own characteristics. The less costly it is for them to provide childcare, the more strongly their own daughters can rely on their support. We will now lay out two mechanisms how grandparental availability to provide childcare increases work-family fit: space and economic activity.

#### Spatial Distance

While some forms of support, like financial support, are not affected by spatial distance, childcare is hands-on and requires physical presence. The closer the grandparents live to their daughters, the less traveling time it takes to get to their daughter’s house, and the less costly it is for them to provide help with childcare. This can enable young women to participate in education or on the labor market. Indeed, proximity to grandparents has been shown to be an important predictor of different fertility related outcomes, such as child birth (Thomese & Liefbroer, 2013; García-Morán & Kuehn, 2017), frequency of grandparental childcare (Knijn & Liefbroer, 2006; Dimova & Wolff, 2008; Thomese & Liefbroer, 2013; Ho, 2015; Zamarro, 2020), and of the daughter’s labor force participation (Compton & Pollak, 2014; García-Morán & Kuehn, 2017). Older adults were also shown to be more likely to move towards their children if they have grandchildren (van Diepen & Mulder, 2008). Sharing a household with their parents has also been shown to increase labor force participation and grandparental childcare provision (Leibowitz et al., 1992; Ogawa & Ermisch, 1996; Sasaki, 2002; Vandell et al., 2003). From this we deduce:

Hypothesis H3: The more grandparents live in close distance to the young mother, the less likely it is that she becomes NEET and the more likely she is to exit NEET.

#### Economic Activity

Within the extended family, cost-benefit calculations like the ones within the nuclear family are made. For instance, as wage growth and human capital investments flatten out with age (Ben-Porath, 1967; Becker, 1981), having grandparents to provide childcare for them would incur lower family-wide opportunity costs than if the mothers themselves stopped working. It might thus be rational for grandparents to reduce working hours and give up labor earnings so that their daughter can invest in her life-time earnings while she is still in the phase of steep wage growth (see Krolikowski et al., 2020). Hence, the availability of grandparents to supply childcare depends on their own employment and living situation (Hank & Buber, 2009; Bratti et al., 2018). Following this logic, we may assume that grandparents who work part-time or who depend less on income from their own labor have lower opportunity costs for the time not spent on the labor market and are therefore more likely to provide childcare. As a result, young mothers could more strongly rely on their support with childcare (Gray, 2005; Hank & Buber, 2009). However, grandparents who themselves need care are less available for childcare. From this follows:

Hypothesis H4a: Young mothers whose parents work part-time, who are retired, or receive social benefits, are less likely to become NEET and more likely to exit NEET than young mothers whose parents are working full-time.

An alternative mechanism is possible as well. Parish et al. (1991) found that employment of nearby kin increases the employment of mothers themselves. They suggested that this is in line with a “culture-of-employment”- hypothesis. From this follows:

Hypothesis H4b: Young mothers whose parents are working, are less likely to become NEET and more likely to exit NEET than young mothers whose parents are not working.

### Formal Childcare

Next to their social support networks, young mothers could also rely on institutions like formal childcare. We consider the availability of formal childcare as a type of institutional resource to increase work-family fit. Childcare facilities must be nearby, have capacity available, be affordable, and be in line with the quality demands of the parents. Spatial distance has been described as the most important factor for choosing a childcare facility (Berkhout et al., 2009) and there are concerns of sufficient availability, especially in rural areas of the Netherlands (Noailly & Visser, 2009). From this follows that:

Hypothesis H5: The more formal childcare institutions are nearby, the less likely it is that young mothers become NEET and the more likely it is that they exit NEET.

### Moderation of Informal Childcare by Formal Childcare

Different welfare regimes and family policies likely influence the need for kin support (Saraceno & Keck, 2010). Several arguments were made in the literature. First, the argument of crowding-out of private transfers by public transfers holds that a strong welfare state reduces intergenerational solidarity (Cox & Jakubson, 1995; Igel & Szydlik, 2011) and that informal care would only be provided in case of a lack of public childcare provision (Künemund & Vogel, 2006). In opposition to this functional understanding of intergenerational solidarity stands the argument of crowding-in. It holds that more expansive welfare provisions complement and stimulate intergenerational solidarity (Daatland & Lowenstein, 2005; Igel & Szydlik, 2011). To reconcile these two hypotheses, the concept of ‘mixed responsibilities’ suggests that formal and informal childcare provisions interact (Attias-Donfut & Wolff, 2000; Igel & Szydlik, 2011; Bordone et al., 2017). For example, while the welfare state provides basic, regular care, the family might concentrate on less time consuming, informal care (Igel & Szydlik, 2011). Indeed, public spending on childcare increases occurrence of childcare by grandparents but decreases its frequency (Igel & Szydlik, 2011). In the Netherlands, childcare by grandparents is used to complement part-time work arrangements and the lack of formal childcare and leave schemes (Portegijs et al., 2006; Igel & Szydlik, 2011; Geurts et al., 2015; Bordone et al., 2017). These hypotheses mostly consider the size of the welfare state, family policies, and childcare expenditure on a national level, although they can be adapted to the local level as well. In our case, availability of childcare facilities nearby should interact with grandparental childcare. We cannot distinguish between the different mechanisms as we do not observe frequency or occurrence of grandparental childcare. However, we can distinguish crowding-out from crowding-in. Hence, we expect that:

Hypothesis H6: The higher the availability of formal childcare nearby, the smaller (crowding-out) or larger (crowding-in) is the relationship of grandparental availability and the probability of young mothers to become and exit NEET.

## Data and Methodology

### Data and Population

We use population-wide register data from the Dutch Social Statistical Database (SSD) (Bakker et al., 2014). For the entire population, we know of the educational enrolment, the monthly economic activities, income, and working hours and merge these into a long data file. Using the encrypted personal identifier, we can link young mothers to their parents, their children, the fathers of their children, and their partners. We then add basic demographic data to each of the personal identifiers. Every person is then linked to an encrypted address identifier which we use to calculate distances between them.

We define our target population in the following way. We start with data on each child born in the Netherlands between 2012 and 2014 to which we merge the personal identifier of their legal mother2. We restrict the sample to women born in the Netherlands who were between 16 and 24 when they had their first child. This yields a population of N = 32,365 young mothers with a median birth year of 1990. We expand the data so that every row is equal to one monthly observation per individual. This person-period file consists of N = 2,726,604 person-month observations beginning 24 months before first childbirth to 60 months after. After list wise deletion of observations with missing values on key variables, our final sample includes N = 31,938 young mothers. Tables 1–4 show descriptive statistics of the variables used in the analysis by actor and domain. The following section describes the operationalization of these variables.

**TABLE 1:** Summary statistics of key variables of young mothers.

Young mothers’ characteristics	Freq.	%; mean (SD)
Household situation		
Single	16,186	50.7
Cohabitating	10,012	31.3
Married	5,740	18
Immigration background		
Dutch	25,210	78.9
Caribbean	508	1.6
Moroccan	1,301	4.1
Surinam	1,381	4.3
Turkish	1,250	3.9
Western	1,474	4.6
Non-Western	814	2.5
Mothers’ activity before birth		
Higher Education	2,435	7.6
NEET	3,686	11.5
Secondary Education and below	1,477	4.6
Vocational Training	8,712	27.3
Working	10,574	33.1
Part-time Work	5,054	15.8
Urbanization		
Non-Urban	13,716	42.9
Urban	10,518	32.9
Very urban	7,704	24.1
Province		
Drenthe	1,015	3.2
Flevoland	1,158	3.6
Friesland	1,481	4.6
Gelderland	3,972	12.4
Groningen	1,383	4.3
Limburg	1,796	5.6
Noord-Brabant	3,628	11.4
Noord-Holland	4,339	13.6
Overijssel	2,363	7.4
Utrecht	2,046	6.4
Zeeland	992	3.1
Zuid-Holland	7,765	24.3
*Age in years*	31,938	21.09 (1.85)
N (Young mothers)	31,938	100

**TABLE 2:** Summary statistics of key variables of partners of young mothers.

Partner characteristics	Freq.	%
Immigration background		
Dutch	12,533	79.6
Caribbean	166	1.1
Moroccan	787	5.0
Surinam	340	2.2
Turkish	829	5.3
Western	754	4.8
Non-Western	343	2.2
Partners activity before birth		
Education	4,016	25.5
Working	10,258	65.1
NEET	953	6.1
Part-time Work	525	3.3
Wage as percentage of partner’s wage		
no income from either	2,771	17.6
no income from YM	3,147	20.0
up to 33%	619	3.9
33% to 66%	2,646	16.8
66% to 100%	2,530	16.1
100% and more	1,013	6.4
no income from Partner	3,026	19.2
N (Partners)	15,752	100

**TABLE 3:** Summary statistics of key variables of grandparents of young mothers’ first child.

Grandparents’ characteristics	Freq.	%
Maternal grandmother matched		
No	891	2.8
Yes	31,047	97.2
If yes: Economic activity		
Part-time	8,976	28.9
Full-time	9,761	31.4
Unemployment/Welfare benefits	3,747	12.1
Sickness/Other benefits	2,783	9.0
Pension	491	1.6
other	5,289	17.0
Maternal grandfather matched		
No	3,346	10.5
Yes	28,592	89.5
If yes: Economic activity		
Full-time	21,478	75.1
Unemployment/Welfare benefits	2,313	8.1
Sickness/Other benefits	2,929	10.2
Pension	979	3.4
other	893	3.1
Paternal grandmother matched		
No	5,934	18.6
Yes	26,004	81.4
If yes: Economic activity		
Part-time	6,791	26.1
Full-time	7,433	28.6
Unemployment/Welfare benefits	2,738	10.5
Sickness/Other benefits	2,355	9.1
Pension	1,457	5.6
other	5,230	20.1
Paternal grandfather matched		
No	7,971	25.0
Yes	23,967	75.0
If yes: Economic activity		
Full-time	16,276	67.9
Unemployment/Welfare benefits	1,813	7.6
Sickness/Other benefits	2,782	11.6
Pension	2,541	10.6
other	555	2.3

**TABLE 4:** Summary statistics of childcare availability variables.

Childcare availability	Freq.	%
Number of grandparents within 3 km radius		
0	7.186	22,5
1	5.627	17,6
2	12.249	38,4
3	2.823	8,8
4	4.053	12,7
Childcare facilities within 3 km radius		
0	1,290	4.5
1–3	6,415	22.3
3+	21,044	73.2

### Monthly activity

Monthly activity is the central data in this study and both basis for dependent and independent variables. It is obtained by merging three datasets from the Dutch register data (SSD) (Bakker et al., 2014). First, we use monthly data on the main economic activity which is defined as the main source of income. Originally, this variable has twelve states: (1) employee, (2) director/major shareholder, (3) self-employed, (4) other self-employed, (5) recipient of unemployment insurance, (6) recipient of welfare, (7) recipient of other social benefits, (8) recipient of illness and disability benefits, (9) recipient of pension, (10) (not yet) pupil/student with income, (11) (not yet) pupil/student without income, (12) other without income. We collapse states 1-4 into “Working”, states 5–9 and 12 into ‘NEET’, and states 10–11 into “Education”. Second, we use a dataset that includes calendar data on registrations in publicly funded education to better differentiate among different types of education. We merge the two datasets, whereas education overwrites other states. We do this because in some cases the two data sets contain different information, for example when students or apprentices earn an income. We argue that in such cases, the defining state is being in education and not earning an income. Primary education, practical education, and secondary education are grouped as “Secondary Education and below”. We distinguish two main types of further education, Vocational Training and Higher Education. Third, we make use of data on employment contracts with which we can further split-up the state of “Working” into full-time and part-time. We define part-time working as less than three full working days per week (24 h). In most cases, parental leave should be included as employment–if contract or income do not change during it. We repeat these data handling steps for the parents of the mother, the current partner (in case one is present), and the parents of the partner. We obtain the monthly activity for all those key actors in the same way. Although we do code the monthly activity for the parents differently than for the young mother and her partner. For the parent generation, we distinguish between Working (and Education as those are only very few), Unemployment/Welfare benefits, Sickness/other benefits, Pension, and other.

### Young Mother’s Variables

#### Event: Enter/Exit NEET

Our main dependent variables are dummy variables scored 1 in the month a woman experiences an event and 0 in all previous months. Based on the monthly activity, we define the following events. The variable “Enter NEET” takes on the value of 1 in the months a young mother becomes NEET for at least 3 months and 0 in case of no change. The variable ‘Exit NEET’ takes on the value of 1 if a young mother starts work or education for at least 3 months and 0 in case of no change. In total, we record 35,677 entries into NEET and 33,400 exits out of NEET. In the Supplementary Appendix, we show that the choice of the event defined as entering a state for 1 month instead of our preferred definition as an entry into a new state for at least 3 months does not change our conclusions.

#### Prior Economic Activity of Young Mothers

Based on the monthly activity data, we create a time-constant variable of the modal activity of the young mother between 24 and 12 months before her first birth. We distinguish NEET, part-time work, full-time work, secondary education, vocational education and training, higher education.

#### Household situation

We link the personal identifiers to the household data set from which we retrieve the variable household type, which we recode into single (1), cohabitating (2), and married (3).

#### Immigration background

We differentiate between persons without immigration background (with both parents born in the Netherlands) and a second-generation immigration background (at least one parent not born in the Netherlands). We distinguish between seven parental origin categories, Dutch, Caribbean, Moroccan, Surinamese, Turkish, Western and Non-Western. Given our research question being primarily focused on kin support, we exclude first generation immigrants because often there are no parents that we can identify.

#### Urbanization and Province

We account for urbanization because the real travel time might differ between rural and urban areas. Urban areas also have a higher density of formal childcare. At the same time, the labor market and education structures are different in urban areas. For this, we distinguish (0) rural from (1) urban and (2) very urban municipalities. In addition, the provinces in the Netherlands differ in size, density, population, economic sectors, geography, and local culture. All these are potential confounders that affect both available support and probability to be NEET. Hence, we control for the provinces in all models.

#### Time

**FIGURE 1:**
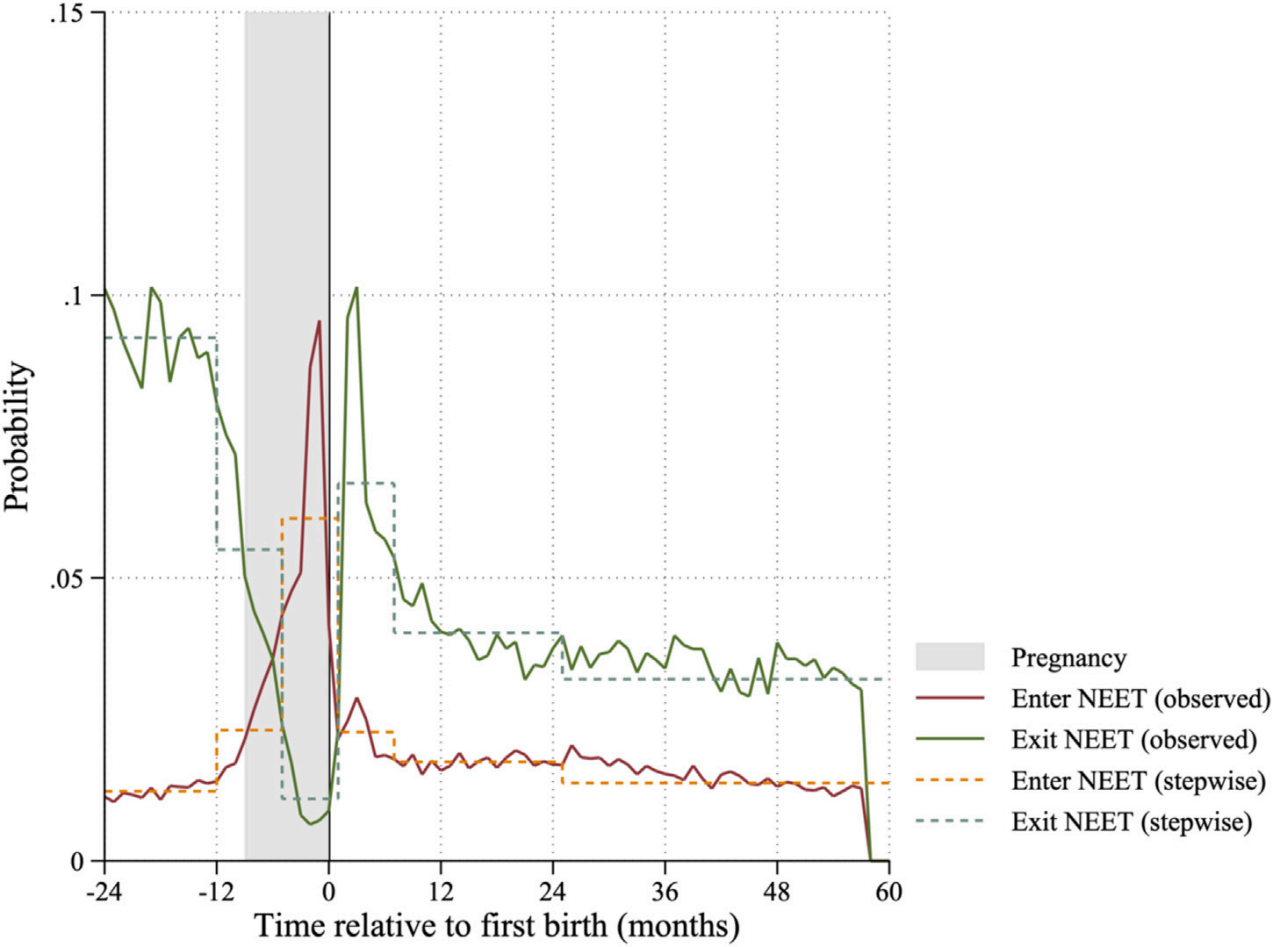
Probabilities and step functions of entering and exiting NEET around the time of first childbirth.

In all models, we include *t*ime relative to the first birth as the piecewise constant baseline hazard. After consideration of the observed monthly hazards shown in Figure 1, we decide on grouping the following months into piecewise constant dummy variables: 24 to 13 months before first birth, 12 to 6 months before first birth, 5 to the month of first birth, 1–6 months after first birth, 7–24 months after first birth, 15– months after first birth. Additionally, we include age (centered at sample mean), age-squared, and the current activities’ spell length in months.

### Partner’s Variables

#### Partner’s Activity Before Birth

Based on the monthly activity data, we create a time-constant variable of the modal activity of the partner between 24 and 12 months before the first birth of the young mother. This variable is coded zero in case there is no partner present. By adding the household situation variable to the model, the coefficient of partner’s activity is to be interpreted among women who are cohabitating or married.

#### Relative Wage

We compare the wage of the young mother to the wage of the partner. Here, we distinguish between several levels of relative wage between less than 33% up to 100% and more. In addition, we distinguish cases in which the young mother, the partner, or both do not earn any wage. In case there is no partner, the variable is coded as “no income from partner”. Hence, the coefficient of relative wage is an additive one and only interpreted if the household situation is cohabitating or married.

### Grandparent Variables

#### Economic Activity of Grandparents

We obtain the monthly activity the same way for all other actors and as described in the paragraph on monthly activity. That is, we obtain a categorical variable distinguishing Education, Full-time working, Part-time working (<24 h per Week, via (S)POLISBUS), NEET, and absence (when no actor can be matched). For grandparents, instead of NEET, we distinguish between receiving between welfare/unemployment, pension, and disability benefits.

#### Distance to Grandparents and Availability in Immediate Vicinity

Based on the household address data, we create address pairs of young mothers and their parents as well as their partner’s parents. We then calculate the distance for all address pairs using the “Distance tool” provided in the remote access environment of Statistics Netherlands. Distance measured as the crow flies correlates strongly with actual travel distance (0.966) and time (0.947) (Rietveld et al., 1999). We then count the number of grandparents living within 3 km of the young mother. In the Dutch context, this corresponds to the surface area of a medium sized town.

### Formal Childcare

We use the number of childcare facilities within 3 km travel distance, which we interpret as a measure of ease of access and availability of formal childcare. We merge this information to each person-period observation via the encrypted address identifier. This measure is only available from 2012 onwards. We use the values from 2012 for 2010 and 2011 and assume that in those years, distances between addresses and childcare facilities have not changed substantially.

## Analytical Strategy

To test our hypotheses, we estimate discrete-time event-history models with repeated events and individual random effects. First, we model the risk for young women to become NEET for at least 3 months during 24 months before until 60 months after the time of first childbirth, given that they were working or in education before. Second, we model the risk of young women who are NEET for at least 3 months to exit the NEET status for at least 3 months.

## Results

The likelihood to enter and exit NEET over time is shown in Figure 1. The likelihood of entering NEET is low before pregnancy, increases during pregnancy, and decreases again after birth to a similar level as before pregnancy. Hence, if young mothers did not become NEET shortly before birth, the likelihood to become NEET in the 5 years after is rather low. We see a similar, mirrored pattern, for the likelihood of exiting NEET. It decreases during pregnancy, and then sharply increases just after birth. After birth, the likelihood to exit NEET decreases again and stays on a lower level than before the pregnancy. Hence, if young mothers do not exit NEET right after birth, the likelihood to exit is lower than before pregnancy. Because these relationships are not easily captured by a functional form, we include time relative to birth as a stepwise function, shown as dashed lines in Figure 1.

**FIGURE 2:**
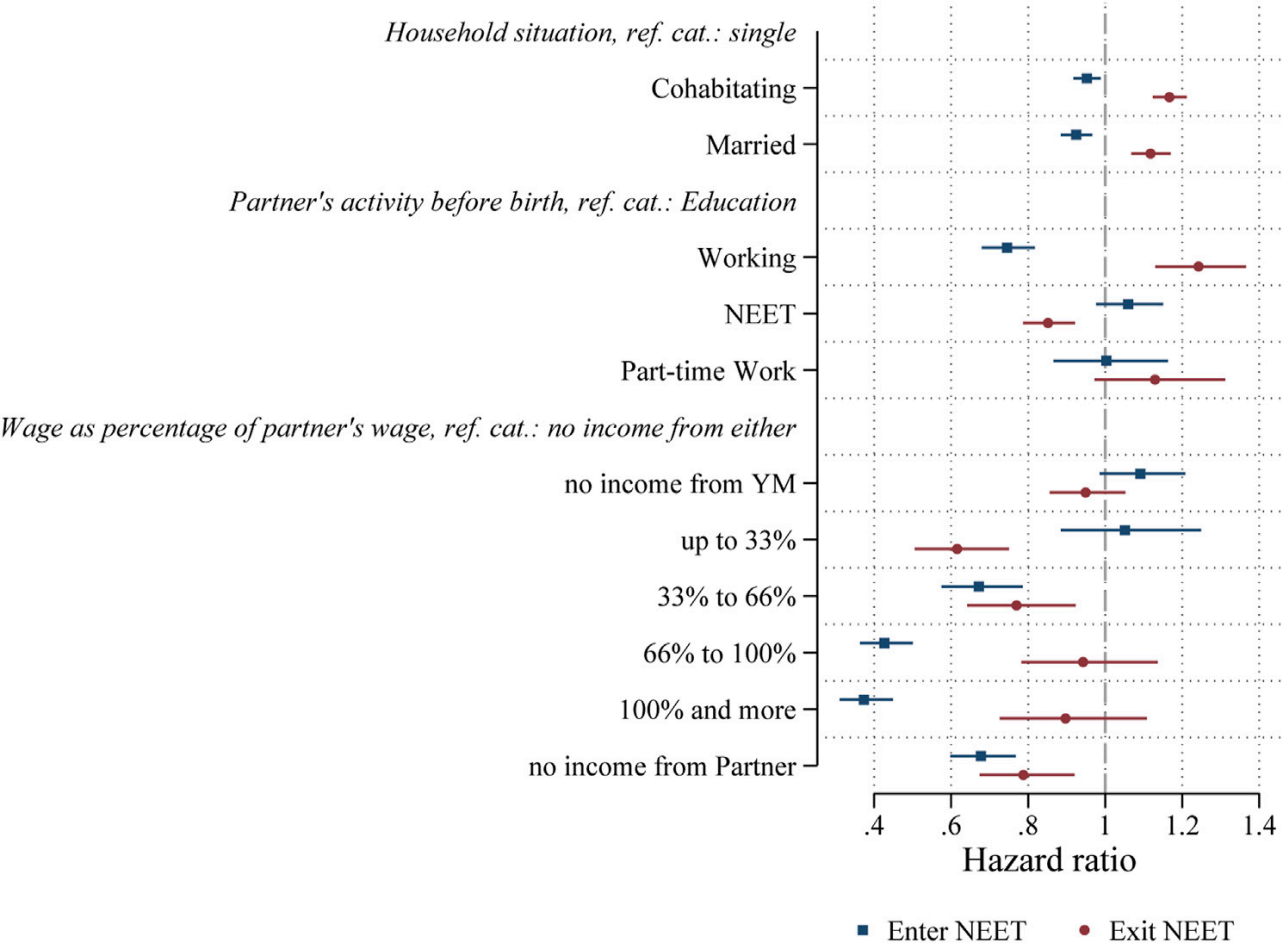
Coefficients of partner characteristics from discrete-time event-history analysis (hazard ratios) of entering and exiting NEET.

We first discuss hypothesis H1 on partner availability. Results are shown in Figure 2 and based on the full model shown in the Table 5. As expected, and shown in Figure 2, young mothers who are currently cohabitating (b = -0.049) or married (b = -0.078) are less likely to become NEET and more likely to exit NEET status (b = 0.154 and b = 0.111, respectively). We therefore accept Hypothesis H1. In addition, if the partner was working full-time, young mothers are less likely to become NEET (b = -0.294) and more likely to exit NEET (b = 0.217). We do not find significant differences for partners who worked part-time, nor for becoming NEET. However young mothers, whose partner was NEET are less likely to exit NEET (b = -0.161).

**TABLE 5:** Discrete-time event history analysis of available support by grandparents, log odds form logistic regression of entry into and exit out of NEET.

	Enter NEET		Exit NEET	
	b	se	b	se
**Partner**				
Household situation, ref. cat.: single				
Cohabitating	−0.049^*^	0.019	0.154^**^	0.019
Married	−0.078^**^	0.023	0.111^**^	0.023
Partner’s activity before birth, ref. cat.: Education^†^				
Working	−0.294^**^	0.047	0.217^**^	0.048
NEET	0.058	0.042	−0.161^**^	0.040
Part-time Work	0.003	0.075	0.122	0.076
Partner’s immigration backgr., ref.cat. Both parents NL-born^†^				
Caribbean	0.023	0.067	0.108	0.069
Moroccan	0.126^*^	0.054	−0.273^**^	0.054
Surinam	0.185^**^	0.050	−0.028	0.051
Turkish	0.143^**^	0.055	−0.063	0.056
Western	0.072	0.037	0.055	0.038
Non-Western	0.020	0.054	0.087	0.052
Wage as percentage of partner’s wage, ref.cat.: no income from either^†^				
no income from YM	0.087	0.052	−0.052	0.053
up to 33%	0.050	0.088	−0.485^**^	0.101
33% to 66%	−0.398^**^	0.080	−0.262^**^	0.093
66% to 100%	−0.852^**^	0.082	−0.059	0.095
100% and more	−0.986^**^	0.094	−0.109	0.108
no income from Partner	−0.390^**^	0.064	−0.239^**^	0.079
**Grandparents**				
Matched grandparents				
Grandmother matched	−0.065	0.041	0.126^**^	0.039
Grandfather matched	−0.099^**^	0.024	0.104^**^	0.024
Father’s mother matched	−0.106^**^	0.028	0.143^**^	0.028
Father’s father matched	−0.058^*^	0.023	0.090^**^	0.023
Number of grandparents within 3km, ref. cat.: no^‡^				
1	0.024	0.020	0.072^**^	0.020
2	−0.105^**^	0.017	0.152^**^	0.018
3	−0.169^**^	0.027	0.144^**^	0.027
4	−0.275^**^	0.026	0.213^**^	0.027
Monthly activity maternal grandmother, ref. cat.: Part-time work^‡^				
Full-time	0.027	0.018	0.032	0.019
Unemployment/Welfare benefits	0.191^**^	0.023	−0.176^**^	0.024
Sickness/Other benefits	0.159^**^	0.025	−0.206^**^	0.026
Pension	0.023	0.049	−0.096	0.050
other	0.108^**^	0.021	−0.129^**^	0.023
Monthly activity maternal grandfather, ref. cat.: Full-time work^‡^				
Unemployment/Welfare benefits	0.158^**^	0.025	−0.171^**^	0.026
Sickness/Other benefits	0.131^**^	0.023	−0.172^**^	0.024
Pension	0.025	0.035	−0.081^*^	0.035
other	0.125^**^	0.039	−0.121^**^	0.040
Monthly activity paternal grandmother, ref. cat.: Part-time work^‡^				
Full-time	0.020	0.021	−0.000	0.022
Unemployment/Welfare benefits	0.175^**^	0.028	−0.179^**^	0.029
Sickness/Other benefits	0.105^**^	0.029	−0.057	0.030
Pension	0.108^**^	0.034	−0.046	0.035
other	0.035	0.024	−0.096^**^	0.025
Monthly activity paternal grandfather, ref. cat.: Full-time work^‡^				
Unemployment/Welfare benefits	0.137^**^	0.029	−0.163^**^	0.031
Sickness/Other benefits	0.105^**^	0.025	−0.121^**^	0.026
Pension	0.046	0.026	−0.061^*^	0.027
other	0.016	0.048	−0.055	0.053
**Individual variables**				
Activity before birth, ref. cat.: VET				
Higher Education	−0.338^**^	0.030	0.408^**^	0.032
NEET	1.365^**^	0.029	−1.319^**^	0.033
Secondary Education and below	−0.048	0.035	−0.279^**^	0.039
Working	0.174^**^	0.063	0.389^**^	0.079
Part-time Work	0.664^**^	0.067	0.314^**^	0.081
Children, ref. cat.: no				
1	−0.180^**^	0.040	−0.421^**^	0.100
2	−0.232^**^	0.047	−0.407^**^	0.102
3+	−0.073	0.076	−0.719^**^	0.110
Immigration backgr., ref.cat. Both parents NL-born				
Caribbean	−0.054	0.051	0.151^**^	0.049
Moroccan	0.339^**^	0.046	−0.194^**^	0.048
Surinam	−0.005	0.036	0.139^**^	0.034
Turkish	0.353^**^	0.050	−0.060	0.051
Western	0.075^*^	0.033	0.028	0.033
Non-Western	0.004	0.044	0.109^*^	0.043
Urbanization, ref. cat.: rural				
Urban	0.061^**^	0.017	−0.073^**^	0.018
Very urban	0.116^**^	0.023	−0.119^**^	0.023
Province, ref. cat.: Drenthe				
Flevoland	−0.063	0.054	0.090	0.052
Friesland	−0.011	0.050	0.066	0.049
Gelderland	−0.144^**^	0.045	−0.011	0.044
Groningen	−0.086	0.052	−0.078	0.052
Limburg	−0.016	0.049	−0.015	0.049
Noord-Brabant	−0.087	0.045	0.067	0.044
Noord-Holland	−0.247^**^	0.046	0.182^**^	0.044
Overijssel	−0.060	0.048	−0.004	0.047
Utrecht	−0.182^**^	0.049	0.096^*^	0.048
Zeeland	−0.176^**^	0.057	0.115^*^	0.057
Zuid-Holland	−0.245^**^	0.045	0.124^**^	0.043
Time relative to first childbirth, ref. cat.: −24 months to −13 months				
−12 to −6 months	0.827^**^	0.022	−0.760^**^	0.032
−5 to 0 months	2.141^**^	0.022	−2.817^**^	0.051
+1 to +6 months	1.432^**^	0.048	−0.534^**^	0.104
+7 to +24 months	1.108^**^	0.046	−0.660^**^	0.104
+25 to +60 months	1.205^**^	0.050	−0.586^**^	0.105
Age (centered)	−0.074^**^	0.005	0.015^**^	0.005
Age squared	−0.013^**^	0.001	−0.004^**^	0.001
Length of current spell	−0.027^**^	0.001	−0.044^**^	0.001
Constant	−4.154^**^	0.066	−1.746^**^	0.065
Individual-level random effect	0.491^**^	0.015	0.337^**^	0.017
Events	35528		33264	
Persons	30905		24040	
Person-months	1891586	824942
ICC	0.130	0.093
-2LL	−161288.047	−121471.407	

a These variables are coded zero for single mothers.

b These variables are coded zero in case of no matched grandparent.

*p < 0.05 **p < 0.01.

Next, in hypothesis H2 we expected that the higher the relative income of the young mother compared to her partner would decrease the likelihood for her to become NEET. We find evidence to suggest that this is the case. The coefficients of the relative wage variable are coded zero in case there is no partner present. Within this group, the higher the share of the young mother’s wage income compared to the partner, the less likely she is to become NEET. Comparing the coefficients, this effect tends to increase with the size of the relative share and is highest in case of income parity or when the young mother earned more than her partner. However, also if the partner did not earn before birth, the likelihood to become NEET is decreased as well. For exiting NEET, the pattern is less clear. We find that having a low relative share of the wage within the relationship is correlated with a decreased likelihood to exit NEET. Note that this is only relevant for young mothers who worked and who have a partner who worked. Within this group, a low share of the income rather than parity, is correlated with a lower likelihood to exit NEET. Overall, we find the results to be in line with the argument made in H2 and therefore accept H2. However, we also find that having no income from the partner is negatively correlated with both, the likelihood to enter NEET and exit NEET. This is a special case of the “100% and more” category, with the addition that the income from the partner is not only much smaller but non-existent. Hence, the finding to “Enter NEET” is in line with the finding that having a higher share of the income is correlated with a lower NEET risk. We tentatively interpret this as evidence for a need to work, that arises from a lack of material support from the partner. In the case of exiting NEET, the context is different because the current activity is different. All the young mothers in this estimation sample are currently NEET. In this case, having a partner who was not earning income while having earned income herself, decreases the risk to exit NEET. Meaning, that once these young mothers do become NEET (against the odds, as the negative coefficient in the “Enter NEET” model shows), they have a lower likelihood to exit NEET. We tentatively interpret this as a lack of support for exiting NEET.

**FIGURE 3:**
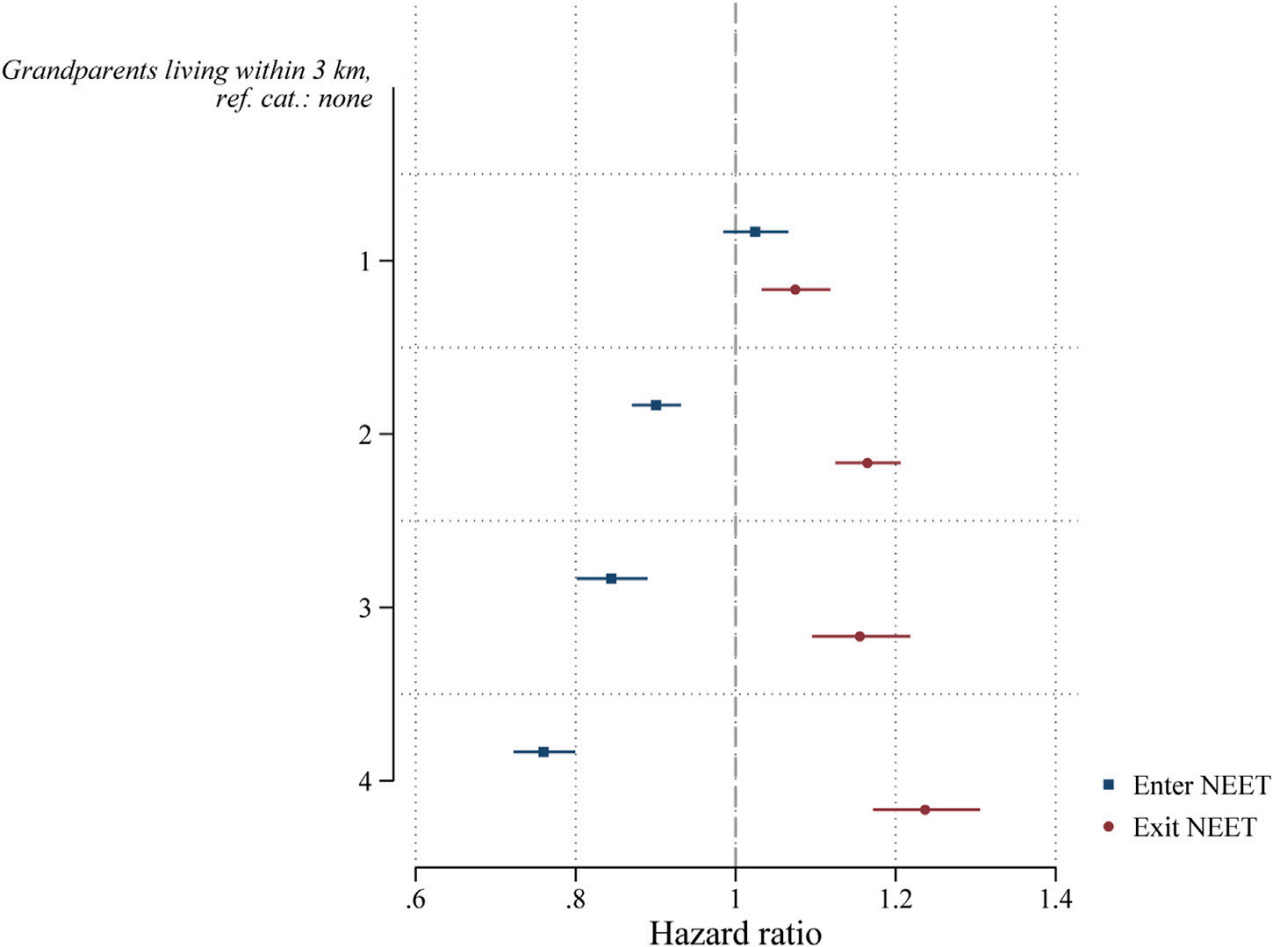
Coefficients of grandparental availability within 3 km from discrete-time event-history analysis (hazard ratios) of entering and exiting NEET.

Figure 3 shows the results to test hypothesis H3 on the number of available grandparents in the immediate vicinity. We expected that the more grandparents live within 3 km of the young mother, the more potential support young mothers can access and thereby decrease the likelihood to become NEET and increase their likelihood to exit NEET status. In both cases we find the expected pattern. First, if either one of the grandparents is matched in the data, which we interpret as being an available source of support, decreases the likelihood of becoming NEET and increases the likelihood of exiting NEET. The only exception is the maternal grandmother; however, this is likely due to the lack of variation in this variable (see Table 3, 97.2% of young mothers have their mother matched in the data). In addition, compared to not having any grandparents nearby, young mothers with two (b = −0.105), three (b = −0.169), or four (b = −0.275) grandparents nearby are less likely to become NEET. We do not find a significantly lower likelihood for one grandparent nearby (b = −0.024). However, these coefficients need to be interpreted in addition to having at least one grandparent matched in the data, and a single grandparent living nearby is likely a case of divorce or death, which is likely to change the family structure itself. For exiting NEET, the pattern is very similar, although here we do find the expected coefficient also for the first grandparent. Compared to not having any grandparents nearby, young mothers with one (b = 0.072), two (b = −0.152), or three (b = −0.144), or four (b = 0.213) grandparents nearby are more likely to exit NEET status. Following this, we accept Hypothesis H3.

Next, we turn to hypothesis 4a and 4 b on the economic activity mechanism of grandparental availability. We do not find significant differences in the likelihood to enter and exit NEET for part-time working grandmothers compared to full-time working grandmothers. In addition, we find that having an unemployed (b = 0.191) or sickness benefits receiving mother (b = 0.159) is correlated with a higher likelihood to become NEET for young mothers. While the result for receiving sickness benefits is in line with the availability hypothesis H4a, the results for receiving welfare benefits are more in line with social class and culture-of-employment arguments which we formulated in hypothesis H4b. For the other family members, the results are largely in line with those of the maternal grandmother.

**FIGURE 4:**
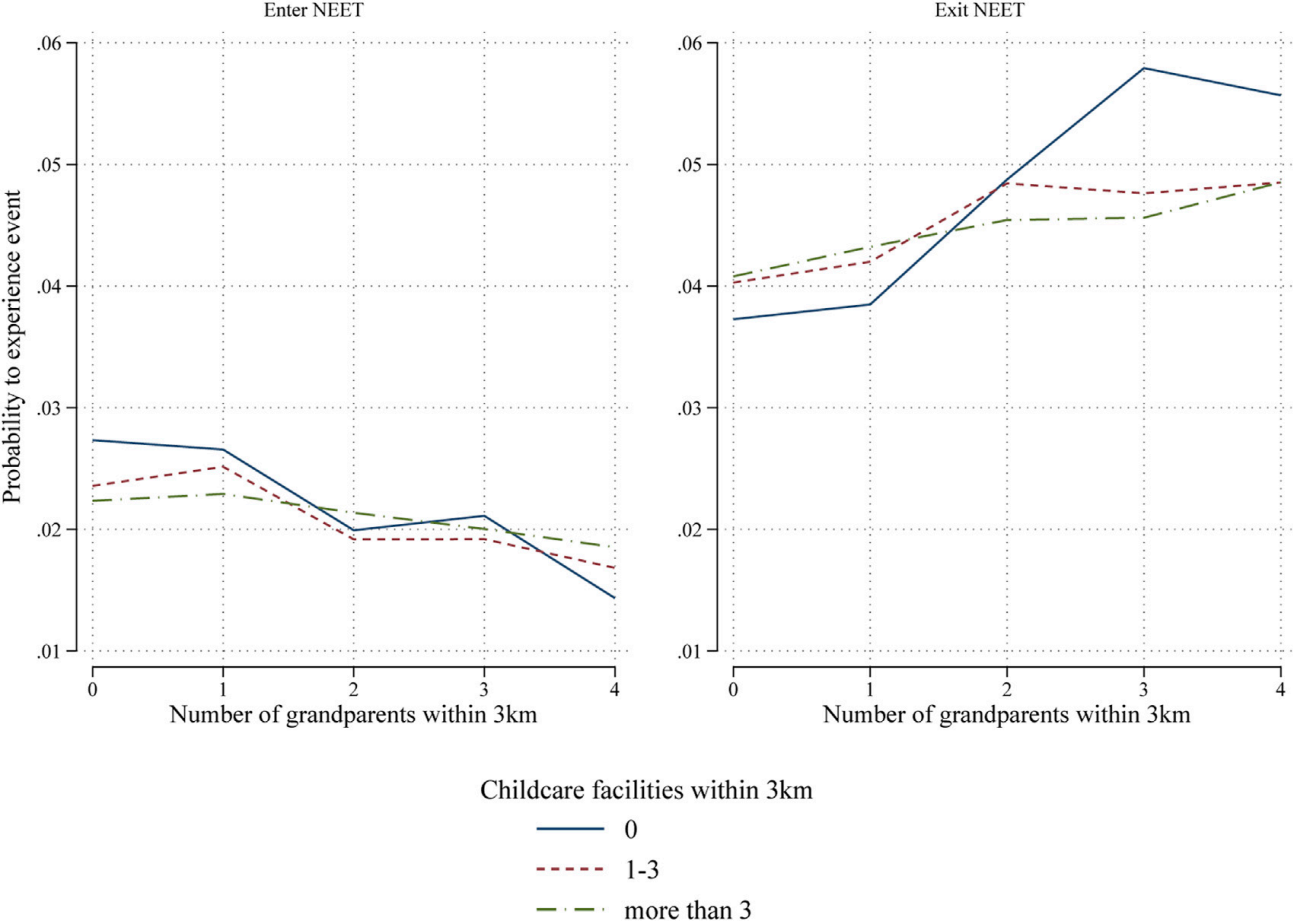
Predicted probabilities of entering and exiting NEET for different levels of available formal and informal childcare support.

Lastly, we expected that the availability of formal childcare would be correlated with a lower likelihood to enter NEET and a higher likelihood to exit NEET. However, we do not find the expected results (shown in the Supplementary Appendix A2). We further expected that the availability of formal childcare moderates the role of informal childcare. Results are shown in Table 6 in line with our expectation, we find that if there are no grandparents nearby, the availability of formal childcare is correlated with a lower likelihood for young mothers to become NEET (b = −0.161 and b = −0.218), although not with a higher likelihood to exit NEET. Vice versa, if there is no formal childcare nearby, having more grandparents nearby translates to a lower likelihood to become NEET and a higher likelihood to exit NEET. We furthermore find some evidence for the crowding-out mechanism of informal childcare. To help interpretation of the interaction, we show them in Figure 4. The horizontal axis shows the number of grandparents within 3 km. The lines represent the predicted probabilities for young mothers to become NEET who live in an area without formal childcare facilities nearby (solid blue), 1-3 childcare facilities nearby (red dashed), and more than 3 facilities nearby (green dash-dotted). As more grandparents are available, all three lines decrease towards the right side of the plot. However, the line for no childcare facilities nearby decreases steeper than the line for 3 or more facilities nearby, meaning that the role of the availability of grandparents becomes less important as the number of formal childcare facilities increases.

**TABLE 6:** Discrete-time event history analysis of formal childcare availability measured as interacted with availability of informal childcare, logistic regression of entry into and exit out of NEET.

	Enter NEET		Exit NEET	
	b	se	b	se
Number of childcare facilities within 3km, ref. cat.: No				
1–3	−0.161^*^	0.063	0.086	0.067
3+	−0.218^**^	0.061	0.100	0.064
Number of grandparents within 3km, ref. cat.: no				
1	−0.031	0.113	0.035	0.129
2	−0.341^**^	0.079	0.301^**^	0.085
3	−0.279	0.170	0.499^**^	0.149
4	−0.690^**^	0.125	0.453^**^	0.142
Interaction terms				
1–3 X 1	0.101	0.120	0.011	0.137
1–3 X 2	0.120	0.086	−0.094	0.093
1–3 X 3	0.059	0.179	−0.311	0.161
1–3 X 4	0.329^*^	0.132	−0.244	0.150
3 + X 1	0.058	0.115	0.029	0.131
3 + X 2	0.293^**^	0.082	−0.180^*^	0.087
3 + X 3	0.162	0.173	−0.373^*^	0.152
3 + X 4	0.489^**^	0.128	−0.258	0.145
Constant	−3.925^**^	0.088	−1.878^**^	0.090
Individual-level random effect	0.494^**^	0.016	0.330^**^	0.017
Events	33180		31292	
Persons	30658		23858	
Person-months	1772469		786175	
ICC	0.131		0.091	
-2LL	−150892.804		−114901.492	

Variables not shown: Time to birth (piecewise constant), Number of children, Mother’s prior economic activity, Immigration background, Age, Age-squared, Length of current spell, Urbanization grade, Province, Partners prior activity, Partner’s immigration background, Relative wage, Grandparental activity.

*p < 0.05 **p < 0.01.

## Conclusions and Discussion

We investigated the role of availability of informal and formal childcare for the economic and educational situation of young mothers in the Netherlands. First and foremost, we found that social support from partners and from grandparents is important to young mothers in the Netherlands and that we can confirm prior evidence from qualitative studies (Sniekers & van den Brink, 2019; Ypeij, 2009). We showed that there are significant associations of availability of informal childcare and the likelihood to become and exit NEET for young mothers. Young mothers with a partner and with more bargaining power in the household are less likely to become NEET and more likely to exit NEET. Young mothers with more grandparents living nearby can more readily rely on their help with childcare. This is in line with previous research showing the importance of geographical distance for the frequency of grandparental childcare (Knijn & Liefbroer, 2006; Dimova & Wolff, 2008; Thomese & Liefbroer, 2013; Ho, 2015; Zamarro, 2020), and the daughter’s labor force participation (Compton & Pollak, 2014; García-Morán & Kuehn, 2017). For grandparents who live nearby, travel time is less of an opportunity cost than for grandparents who live further away. Their childcare can be used more efficiently and more spontaneously, aiding young mothers in navigating the challenges they face in education and on the labor market. We also found that the availability of formal childcare does not have the overall strong relationship with NEET risks that we expected. This is likely because a good spatial coverage of childcare facilities exists. Unfortunately, we did not have access to data on the actual usage of formal childcare, nor on the capacity of the facilities. However, in case of no grandparents around to help, young mothers do indeed rely on formal childcare and vice versa the role of grandparents is strongest when no childcare facilities are around. This is in line with previous evidence on interaction of informal childcare and formal childcare (Attias-Donfut & Wolff, 2000; Künemund & Vogel, 2006; Igel & Szydlik, 2011; Bordone et al., 2017). Unfortunately, we did not have access to data on the actual usage of formal childcare, nor on the capacity of the facilities. Another aspect of formal childcare that we could not directly address are its costs. We speculated in the introduction, that the fact that childcare subsidies are only paid if both parents are working or in education might be problematic for young mothers. While we do find that being NEET and having a partner who is NEET is negatively correlated with exiting NEET, the lack of access to subsidies in these cases is just one of multiple causal pathways. Without usage data, or an external source of variation in access to subsidies it is difficult to properly disentangle these.

Naturally, our approach using register data has its strengths but also some shortcomings. While we do not have to rely on potentially selective survey data, we cannot directly measure the actual provision of childcare. Therefore, our results can only be interpreted as availability and not as provision. Of course, the presence of grandparents does not mean that every young mother also uses this access for childcare. However, this also means that the “true effect” of grandparental provided childcare is likely larger than the coefficients of presence that we observe. Hence, our analyses are to be interpreted like intention-to-treat effects (Arpino et al., 2014), with the important caveat that we only control on observable characteristics and therefore explicitly refrain from a causal interpretation. While the application of register data also helped us to minimize the risk to overlook small groups and to eliminate common issues with survey data such as panel attrition, our data did also lack some important variables to further interpret our findings. Future research should therefore try to combine survey and register data.

## Data Availability

The data analyzed in this study is subject to the following licenses/restrictions: Data from Statistics Netherlands is available only to registered users. Requests to access these datasets should be directed to microdata@cbs.nl.

## Footnotes

1 Although not all of the gender wage gap can be explained with the motherhood penalty (Combet & Oesch, 2019).
