# Supplementary material for: How Young Mothers Rely on Kin Networks and Formal Childcare to Avoid Becoming NEET in the Netherlands
Source: Front Sociol. 2022 Jan 27;6:787532. doi: 10.3389/fsoc.2021.787532 (PMC8829039; doi:10.3389/fsoc.2021.787532)
Supplement: Supplementary file 1 [file DataSheet1.docx]

**Appendix**

Appendix A1: Literature overview of grandparental childcare and mothers' labour market activities

| **Author(s)** | **Year** | **Countries** | **Data type** | **Age of**  **mothers** | **Years**  **studied** | **Sample size (mothers)** | **Methods** | **DV** |
| --- | --- | --- | --- | --- | --- | --- | --- | --- |
| Del Boca | 2002 | IT | panel | 36 | 1991-1995 | 1708 | Fixed-effects panel regression; ITT (grandparent alive) | Working at time of interview |
| Dimova & Wollf | 2008 | FR | cross-sectional  survey | 33 | 2002/2003 | 2202 | Probit family random effects & family fixed effects logit & 2SLS with endogenous grandchildcare | LFP at time of interview |
| Dimova & Wollf | 2011 | AT, CHE, DE, DK, ES, FR, GR, IT, NL, SWE | cross-national  panel (cross-sectional) | 36 | 2004 | 2317 | Probit and family-fixed effets | LFP at time of interview |
| Aassve, Arpino & Goisis | 2012 | BG, DE, FR, GEO, HU, NL, RU | cross-sectional  survey | 20-55 | ca. 2004 | ca. 1000 per county | Probit IV (maternal grandmother alive & number of siblings of mother) | LFP at time of interview |
| Posadas & Vidal-Fernandez | 2013 | US | panel | 18 - 49 | 1979-2006 | 14659 | FE & IV (grandmother's death) | LFP |
| Compton & Pollak | 2014 | US | cross-sectional  survey & census data | 25-60 & 18-45 (military wives) | 2000 (military wives) | ca. 2500 (married women), 1637 (unmarried women), 10578 (military wives) | Probit/Tobit IV (proximity) | Positive work hours/weekly working hours, LFP/employment |
| Aparicio Fenoll & Vidal-Fernandez | 2014 | IT | cross-sectional  survey | 20-40 | 1998-2009 (three waves) | 3.612 | TS2SLS IV (changes in retirement eligibility) | LFP at time of interview |
| Arpino et al | 2014 | IT | cross-sectional  survey | 37 | 2003 | 3852 | 2SLS IV (1-4 grandparents alive) | LFP at time of interview |
| Compton | 2015 | CAN | cross-sectional  survey | 45-60 | 2007 | ca. 3000 | Probit(Work), Tobit(Work hours) | Work/Work hours |
| Bratti, Frattini & Scervini | 2018 | IT | rotating panel | 20-49 (mean 37) | 1993-2006 | 8402 | LPM; ITT: retirement eligibility | LFP in a given year |
| Zamarro | 2020 | AT, BE, DE, DK, ES, FR, GR, IT, NL, SWE | cross-national  panel (cross-sectional) | 34 | 2004 | 1452 | Probit IV (retirement eligibility) | LFP at time of interview |
| Aparicio Fenoll | 2020 | AT, BE, CHE, CZ, DE, DK, ES, FR, GR, IRL, IT, LUX, NL, PL, PT, SLO, SWE | cross-national panel | 23-50 | 2004-2015 | 25,794 | 2SLS IV (changes in retirement eligibility); ITT | LFP at time of interview |

Appendix A2: Discrete-time event history analysis of formal childcare availability, logistic regression of entry into and exit out of NEET.

|  | Enter NEET |  | Exit NEET |  |
| --- | --- | --- | --- | --- |
|  | b | se | b | se |
| *Number of childcare facilities within 3km, ref. cat.: No* | |  |  |  |
| 1-3 | -0.065 | 0.037 | 0.017 | 0.039 |
| 3+ | -0.039 | 0.037 | -0.003 | 0.039 |
| Constant | -4.085^**^ | 0.075 | -1.783^**^ | 0.076 |
| Individual-level random effect | 0.495^**^ | 0.016 | 0.331^**^ | 0.017 |
| Events | 33180 |  | 31292 |  |
| Persons | 30658 |  | 23858 |  |
| Person-months | 1772469 |  | 786175 |  |
| ICC | 0.131 |  | 0.091 |  |
| -2LL | -150916.657 |  | -114909.461 |  |

Source: Statistics Netherlands, own calculations

Based on full model, variables not shown: Partner characteristics, Grandparent characteristics, Young mother’s characteristics

* p <0.05 ** p <0.01

Appendix A3: Coefficients from discrete-time event-history analysis of main variables of interest, logistic regression of entry into and exit out of NEET with two different operationalizations of event duration.

Source: Statistics Netherlands, own calculations

Appendix A4: Coefficients of grandparental availability within 10 km, discrete-time event-history analysis (hazard ratios) of entering and exiting NEET.

Source: Statistics Netherlands, own calculations
